# CROSS-CULTURAL ADAPTATION AND VALIDATION OF THE JAPANESE TRANSLATION OF THE FUGL-MEYER ASSESSMENT FOR UPPER AND LOWER EXTREMITY SENSORIMOTOR FUNCTION AFTER STROKE

**DOI:** 10.2340/jrm.v57.43350

**Published:** 2025-08-05

**Authors:** Yukitaka TOMODA, Toru NAGAO, Tomoko UCHIDA, Hiroki SATO, Masatoshi OONISHI, Takayuki OKAMOTO, Maya TAJITSU, Margit ALT MURPHY

**Affiliations:** 1 Graduate School of Health Science, Kobe University, Kobe, Japan; 2 Department of Rehabilitation, Kosei Hospital, Kobe, Japan; 3 Graduate School of Sustainable System Sciences, Osaka Metropolitan University, Osaka, Japan; 4 Department of Health and Rehabilitation, Institute of Neuroscience and Physiology, Sahlgrenska Academy, University of Gothenburg; 5 Department of Clinical Neuroscience, Institute of Neuroscience and Physiology, Sahlgrenska Academy, University of Gothenburg, Sweden

**Keywords:** assessment, cross-cultural validation, Fugl-Meyer Assessment, motor function, sensory function, stroke, translation

## Abstract

**Objective:**

To develop an official translation of the original Fugl-Meyer Assessment of upper and lower extremity sensorimotor function into Japanese by following a standardized cross-cultural adaptation process to ensure conceptual, linguistic, and semantic validity.

**Design:**

Cross-cultural translation/validation.

**Subjects/Patients:**

Seven Japanese clinical experts and an external expert developed the translation. The pilot study included 10 participants with stroke.

**Methods:**

Following the Translation and Cross-Cultural Adaptation of Objectively Assessed Outcome Measures, the Fugl-Meyer Assessment was forward and backward translated and reviewed by an expert group and an external expert familiar with the original scale. The translation was tested in a pilot study with 10 patients with hemiparetic stroke to identify problematic items in intra- and inter-rater agreements.

**Results:**

Sufficient intra- and inter-rater agreements (> 70% agreement) were reached for all items, and only 2 sensation items showed systematic disagreement. These results were incorporated to refine the translation and ensure conceptual, semantic, and linguistic equivalence with the original Fugl–Meyer Assessment.

**Conclusion:**

The culturally validated upper and lower extremity Fugl-Meyer Assessment supports the Japanese rehabilitation field and promotes international collaboration by improving the unified assessment of sensorimotor function in individuals with stroke.

## LAY ABSTRACT

Regaining movement and coordination after stroke is essential for patients to live independently. The Fugl-Meyer Assessment is a key tool used worldwide to measure sensorimotor function. As no official Japanese version of the original scale was available, a standardized process was used to translate and culturally adapt the Fugl-Meyer Assessment for Japanese patients and context. When tested in 10 patients with stroke, the translated version showed good agreement both within and between the raters. This new Japanese Fugl-Meyer Assessment allows healthcare professionals in Japan to assess and track patient progress in a standardized manner. This ensures effective treatment planning, comparison of patient outcomes between sites, and promotion of international collaboration.

Stroke is the second leading cause of death worldwide and the third leading cause of mortality and disability, combined (1). It frequently leads to motor dysfunction, which significantly contributes to long-term disability among survivors and substantially affects their ability to engage in daily activities, thereby reducing social participation and profoundly affecting both patients and their families (2). Additionally, the substantial global and financial burden of stroke requires a comprehensive understanding of policies and healthcare strategies (3). Therefore, stroke not only leads to functional impairments and limitations in social participation but also underscores the importance of rehabilitation efforts to address long-term challenges (4).

Treatments are not universally applicable to all patients with stroke; therefore, the target populations must be clearly defined in clinical trials (2). Standardized assessments are essential for accurately evaluating impairments and predicting rehabilitation outcomes and should be easy to administer, score, and interpret, cost-effective, portable, and capable of capturing changes (2, 4).

The original Fugl-Meyer Assessment (FMA) was developed by Fugl-Meyer et al. in 1975 in English and Swedish based on the Brunnstrom recovery stages (5). The FMA is a comprehensive quantitative assessment method designed to evaluate motor function, sensation, passive range of motion, and joint pain in patients with hemiparesis after stroke (5). The reliability and validity of the FMA is excellent for the motor domains, and acceptable for non-motor domains (6–13). The motor domain of the FMA is one of the most widely used assessment tools for post-stroke hemiparesis worldwide (6, 14) and is recommended as a core measure for stroke recovery trials (15). The original FMA has been translated into several languages, including Spanish (16), Italian (17), Danish (18), Korean (12), Urdu (19), Romanian (20), and Czech (21), using standardized cross-cultural adaptation guidelines (22, 23), which ensure that the translated versions are linguistically validated for these languages.

Amano et al. (24) translated the upper extremity items of a modified FMA version presented in Platz et al. (25) into Japanese, whereas Nakazono et al. (26) translated the lower extremity items of a modified FMA version presented in Sullivan et al. (27). These modified versions differ from the original FMA in item definitions, administration procedures, and scoring rules, undermining consistency in clinical assessment and preventing valid comparisons of outcomes.

Thus, the original FMA has not yet been officially translated into Japanese using standardized cross-cultural adaptation guidelines. Therefore, there is a necessity to create a linguistically validated Japanese version of the original FMA to enable unified use nationally and accurate comparison of research findings internationally. This will empower international collaborative research and potentially bring significant benefits to the field of Japanese rehabilitation.

We aimed to perform an official translation of the original FMA of upper and lower extremity into Japanese by following a standardized cross-cultural translation and validation process to ensure conceptual, linguistic, and semantic coherence in the assessment of individuals with stroke.

## METHODS

### Study design

**Fig. 1:**
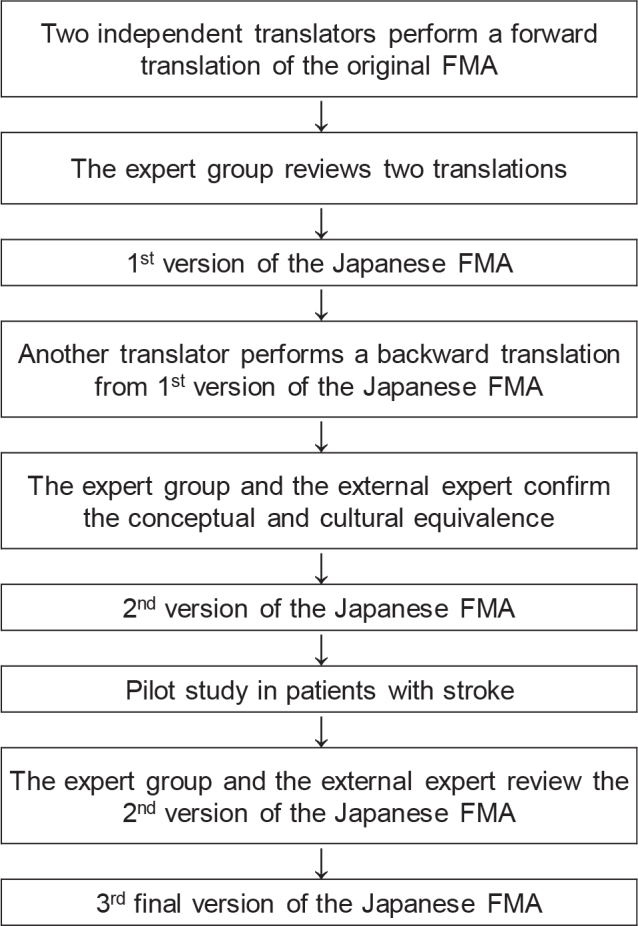
Translation process.

In this study, we adhered to the eight-step procedure for the Translation and Cross-Cultural Adaptation of Objectively Assessed Outcome Measures (TCCA-OAO) (22), a standardized method widely used for developing linguistically validated translated versions, as outlined in previous studies (16, 18). The entire translation process is illustrated in Fig. 1. To ensure the highest quality translation, a rigorous multistep translation process was used, including translation, revision, and drafting of the final version of the Japanese FMA. During the translation and cultural verification process, forward and backward translations were performed by bilingual translators, and the expert group, in collaboration with an external expert user familiar with the original FMA (a physiotherapist with 25 years of clinical and research experience in stroke rehabilitation and in the use of the FMA), verified the conceptual, semantic, and linguistic equivalence between the original FMA and the Japanese version of the original FMA. To detect any discrepancies that could affect understanding, interpretation, cultural applicability, or scoring, a pilot study was conducted with 2 physiotherapists at Kosei Hospital using the 2nd version of the Japanese FMA.

### The Fugl-Meyer Assessment

The original FMA is a comprehensive observational rating assessment tool for evaluating the sensorimotor function in patients with hemiparesis after stroke (5). The items were rated using a 3-point ordinal scale (0=cannot perform, 1=partially performs, 2=performs fully) with the exception of reflex items, which are assessed only as 0 or 2. The assessment of upper extremity motor function has a maximum score of 66 points and includes 4 subscales: upper extremity (0–36 points), wrist (0–10 points), hand (0–14 points), and coordination/speed (0–6 points). The motor assessment for the lower extremities has a maximum score of 34 points and includes the following 2 subscales: lower extremities (0–28 points) and coordination/speed (0–6 points). Additional non-motor domains include H. sensation (0–24 points), I. passive joint range of motion (0–44 points), and J. joint pain (0–44 points) in both the upper and lower extremities.

### Forward translation and drafting of the 1st version of the Japanese Fugl-Meyer Assessment

Before creating the Japanese version of the original FMA, permission to translate the original FMA was obtained from the curator at the University of Gothenburg. The expert group comprised members who were proficient in English and knowledgeable of the original FMA. This group included 2 physiotherapists (17 and 10 years of clinical experience in stroke rehabilitation), 4 occupational therapists (25, 20, 12, and 10 years of clinical experience in stroke rehabilitation), and 1 physiotherapist who also served as a forward translator (7 years of clinical experience in stroke rehabilitation).

Second, 2 forward translators (one of whom was a member of the expert group) native to Japanese and proficient in English were employed by the translation companies. The translators were informed of the purpose of the study. Each translator independently created a draft of the Japanese translation of the original FMA, resulting in 2 forward translations that emphasized a conceptual translation approach grounded in the original FMA.

Third, the expert group rigorously verified the grammar and format of both translations, reviewed each draft in comparison with the original FMA, and discussed it until a consensus was reached. Consequently, the merged draft was produced as the 1st version of the Japanese FMA.

### Backward translation and drafting of the 2nd version of the Japanese Fugl-Meyer Assessment

First, a backward translator native to Japanese and proficient in English was employed by a translation company. The translator was informed of the purpose of the study. The translator then independently created a back-translated version.

Second, the expert group, in collaboration with an external expert, reviewed grammatical and semantic discrepancies between the original FMA and the back-translated version. Based on this feedback, any inconsistencies identified were evaluated for conceptual and cultural equivalence. The expert group discussed this until a consensus was reached, which resulted in the completion of the 2nd version of the Japanese FMA.

### Pilot study

A pilot study was conducted following previous studies (16, 18) to verify the intra- and inter-rater agreements of the 2nd version of the Japanese FMA in 10 patients with hemiparesis after stroke. The raters were 2 physiotherapists with 9 years of clinical experience working at Kosei Hospital. Both raters underwent short training on administration and scoring of the FMA by watching the instructional videos (https://www.gu.se/en/neuroscience-physiology/fugl-meyer-assessment) and applying the assessment in clinical practice prior to pilot study assessments. The inclusion criteria were as follows: first-time stroke with hemiparesis due to cerebral infarction or haemorrhage, > 180 days post-stroke, and age between 18 and 90 years. The exclusion criteria were as follows: Mini-Mental State Examination-Japanese version (MMSE-J) (28) score < 23, extremity amputation, aphasia or psychiatric disorders affecting daily life, severe joint pain precluding study participation, significant visual or hearing impairment, and severe terminal or uncontrollable conditions affecting participation. This study was approved by the Ethics Committee of the Graduate School of Health Sciences at Kobe University (approval number: 1133-5). All the participants provided written informed consent.

Participants’ age, sex, stroke type, modified Rankin Scale score, and MMSE-J score were recorded as background information. The FMA was administered at baseline and repeated 1 week after the first assessment. The rater who performed the first assessment served as the observer during the second assessment, whereas the observer from the first measurement served as the rater for the second assessment. Both raters conducted their assessments independently without any communication during or after the evaluation. The observer observed the rater’s assessment and asked the instructive rater to repeat some items, if necessary.

For the statistical analyses of intra- and inter-rater agreements of the 2nd version of the Japanese FMA, the rank-based statistical methodology (Svensson’s method) was used (29). This method is specifically designed for ordinal data and allows for the evaluation of both systematic and non-systematic disagreements at the item level. Intra- and inter-rater agreement > 70% was considered acceptable, and > 90% was considered excellent (30). To assess systematic disagreement, the relative position (RP), indicating whether 1 rater tended to score higher or lower than another, and the relative concentration (RC), indicating whether scores by 1 rater were more centralized on the scale than another, were evaluated (29, 31). Both RP and RC range from –1 to 1, with 0 indicating no difference between raters (29, 31). The sample size was set at 10 participants based on previous studies (16–18), and a 95% confidence interval (CI) was calculated.

### Drafting of the 3rd version of the Japanese Fugl-Meyer Assessment

To determine whether mistranslation was a factor, items with discrepancies observed in the pilot study along with the raters’ feedback were reviewed. If the expert group identified potential mistranslations, the discussions continued until a consensus was reached. Where a consensus could not be reached, the FMA and backward translation were reviewed, and the 2nd version of the Japanese FMA and the pilot study were reconducted. The agreed 3rd version of the Japanese FMA was reviewed by an external expert for final approval.

## RESULTS

### The 1st version of the Japanese Fugl-Meyer Assessment

The expert group identified several discrepancies in the 2 forward translations of the original FMA. The discrepancies occurred in sections where the instructions were not presented as fully structured sentences but rather as segments. Although the equivalence of word meanings was confirmed in both forward translations, there were instances where the overall meaning of the sentences differed. The expert group discussed these points meticulously and completed the 1st version of Japanese FMA.

For reflex activity, the phrase “can be elicited” in the original FMA was translated as “誘発される” (be elicited) to clarify that tendon reflexes are triggered by the examiner’s action. In the context of synergies, “Volitional movement within synergies” was translated as “共同運動” (synergies), “Volitional movement mixing synergies” as “共同運動を伴う随意運動” (voluntary movement with synergies), and “Volitional movement with little or no synergy” as “共同運動から分離した随意運動” (voluntary movement separated from synergies). Regarding normal reflexes, the term “Lively” was translated as “軽度亢進” (mildly increased) to describe a tendon reflex without pathological significance, ensuring consistency and accuracy in the terminology. These adjustments were made to ensure clarity in ranking while preserving the original intentions.

### The 2nd version of the Japanese Fugl-Meyer Assessment

The expert group and external expert identified several discrepancies when comparing the back translation with the original FMA. These discrepancies were discussed and resolved to complete the 2nd version of the Japanese FMA. For reflex activity, the phrase “can be elicited” in the original FMA had been changed to “elicited” in the back translation, to explain the passive meaning. This was translated to “誘発可能” (can be elicited) in the 2nd version of the Japanese FMA to include the sense of “can”. Discrepancies were also observed in the 3 expressions related to synergies. “Volitional movement within synergies” in the original was changed to “Synergies” in the back translation, omitting “Volitional movement”. This was translated to “随意運動 (共同運動パターン)” (Volitional movement [synergy pattern]) to adequately convey that volitional movement occurs with synergies. For “Voluntary movement mixing synergies” changed to “Voluntary movement with synergy”, this was translated to “随意運動( 分離運動が一部出現)” (Volitional movement [separated from synergy]) to accurately convey the meaning of “mixing synergies”. For “Voluntary movement with little or no synergy” changed to “Voluntary movement separated from synergy”, this was translated to “随意運動(正常、もしくはほぼ正常)” (Voluntary movement [Almost or completely deviated from synergy]) to include the notion of “little or no synergy”. Discrepancies in normal reflexes were found in 2 instances. “Hyper” and “lively” in the original FMA were changed to “increased” and “mildly increased”, respectively, in the back translation. “Hyper” was translated to “著明に亢進” (markedly increased), and “lively” was changed translated to “亢進” (increased) to better convey its pathological significance.

### Pilot study

Ten patients with chronic stroke expressed their willingness to participate and met the set inclusion and exclusion criteria. The average age of the participants was 62.4 ± 8.9 years, including 3 females, and the average MMSE-J score was 29.2±1.3 (Table I). The sample included participants with a varying level of sensorimotor function according to the baseline FMA scores (Table I). The intra- and inter-rater agreements for the 2nd version of the Japanese FMA showed agreement above 90% for most items, whereas some items ranged from 70% to 80% (Tables SI and SII). All the agreements were considered acceptable. Systematic disagreements in RP (–0.3; 95% CI –0.57 to –0.03) were observed for intra-rater agreement in 2 light touch items. Three out of 10 raters assigned lower scores during the second assessment than during the first. No systematic disagreements were found in FMA motor items.

**Table I:** Demographic and clinical characteristics of the participants in the pilot study

No.	Age	Sex	Stroke type	Lesion side	Fugl-Meyer Assessment – UE				Fugl-Meyer Assessment – LE				mRS	MMSE-J
					Motor (0–66)	Sensory (0–12)	ROM (0–24)	Pain (0–24)	Motor (0–34)	Sensory (0–12)	ROM (0–20)	Pain (0–20)		
1	64	F	CI	Right	65	12	24	24	27	12	20	20	1	30
2	74	M	CI	Right	16	10	14	24	21	12	12	20	4	27
3	69	M	CI	Right	52	10	20	21	23	9	15	20	4	28
4	65	M	CI	Left	25	11	15	23	28	11	19	20	2	30
5	76	M	CI	Left	63	10	23	24	25	10	20	20	2	27
6	50	M	ICH	Right	59	12	21	21	21	12	20	20	2	30
7	60	F	CI	Left	61	12	22	21	28	12	19	20	2	30
8	59	F	ICH	Right	63	12	24	24	24	12	20	20	2	30
9	55	M	CI	Right	64	11	24	24	26	10	20	20	2	30
10	52	M	ICH	Right	16	9	23	22	20	12	20	20	3	30

UE: upper extremity; LE: lower extremity; mRS: modified Rankin Scale; ROM: range of motion; MMSE-J: Mini Mental State Examination-Japanese; CI: cerebral infarction; ICH: intracerebral haemorrhage.

### Drafting of the 3rd version of the Japanese Fugl-Meyer Assessment

Based on statistical analyses of discrepancies and feedback from raters in the pilot study, the expert group created a 3rd version of the Japanese FMA. For instance, the “Position sense” item in the sensory domain of the original FMA was translated to “深部感覚” (Deep sensation) to include both “Kinaesthesia” and “Position sense”, as the FMA measures “Kinaesthesia” first and, if necessary, uses imitation to measure “Position sense”. The evaluation criteria for “Deep sensation” from the original were translated as follows: “< 3/4 correct or absence” was translated to “3/4 未満の正答” (< 3/4), “3/4 correct or considerable difference” was translated to “3/4 正答” (3/4 correct), and “100% correct, little or no difference” was translated to “100% 正答” (100% correct). After these revisions, the 3rd version of the Japanese FMA was sent to an external expert for final review. Following a rigorous review, the final version was formally recognized as the Japanese FMA.

## DISCUSSION

In this study, we used a rigorous multistep translation process, including forward and backward translations, in accordance with the TCCA-OAO guideline (22) for translation of clinical observational assessment scales. The thorough translation process, in collaboration with the curator of the original FMA, secured conceptual, semantic, and linguistic equivalence between the original FMA and the Japanese FMA.

The results of this study provide new opportunities for physiotherapists, occupational therapists, physicians, and researchers in the field of stroke rehabilitation to compare patient outcomes in terms of FMA between clinical and research centres, promoting both national and international collaboration (2, 16, 17). This standardization ensures that rehabilitation practices are based on linguistically validated assessments, thereby contributing to the development of better assessment protocols and treatment strategies for patients with stroke. The Japanese FMA translation is publicly available and can be accessed by anyone (https://www.gu.se/en/neuroscience-physiology/fugl-meyer-assessment).

The current Japanese FMA was translated from the original FMA, in contrast to previous translations by Amano et al. (24) and Nakazono et al. (26), which used modified versions published by Platz et al. (25) and Sullivan et al. (27), respectively. For example, the protocol of Platz et al. allowed assistance in obtaining the starting position of the elbow in shoulder flexion and utilized a different starting position for the arm in the coordination/speed item. The protocol published by Sullivan et al. modified starting positions for several items throughout the scale, did not require a full range of wrist extension/flexion for score 2, used altered hand grasp positions, did not perform the coordination/speed items with closed eyes, and also used altered scoring for the coordination/speed section (32). The existence of multiple modified versions could affect the comparison of research data and the development of clinical guidelines (17).

We evaluated the pre-final translated version in 10 individuals with post-stroke to identify potential problematic items that could be a source for discrepancies between raters. Previous translations of the FMA, utilizing similar methodology, reported intra- and inter-rater agreements ranging from 40% to 100% (16–18, 20). In our pilot data, intra- and inter-rater agreement ranged from 70% to 100%, which was considered acceptable. In addition, only 2 sensation items showed systematic disagreements and no systematic disagreements were detected for the motor items. In previous larger studies, the FMA upper and lower extremity sensation domains showed excellent interrater reliability in terms of intra-class correlation coefficients (ICC > 0.90) (12, 13), but low agreement at item level for light touch items (weighted kappa ranging from 0.30 to 0.55) (13) and lower ICC values for the lower extremity passive ROM and pain domains (ICC < 0.70) (13), respectively. Subsequently, additional careful revision of the sensation items was performed to further enhance the quality of the Japanese translated version.

Indeed, further evaluation of the intra- and inter-rater reliability in larger sample of Japanese patients with stroke is recommended. The pilot testing included only patients in the chronic stage of stroke, which is a limitation. The chronic stage of stroke was, however, deliberately selected to minimize a potential patient-induced bias, as patients in the acute and subacute stage might be more tired or present a more unstable motor performance compared with the chronic stage. Another limitation was that the raters of the pilot study had only a limited experience of the FMA. In a larger reliability evaluation, it is important to minimize this bias. However, in clinical practice, where therapists with varying levels of experience will apply the FMA, the use of the novice raters can be justified in detecting potential areas of misunderstandings.

In conclusion, we successfully developed the Japanese FMA by adapting the original FMA to the Japanese culture using standardized cross-cultural guidelines and validating its linguistic accuracy, thereby expanding its global accessibility. These findings not only create new opportunities for clinicians and researchers to compare FMA-based patient outcomes between Japan and other countries, fostering national and international collaboration, but also contribute to the standardization and broader adoption of the Japanese FMA in clinical settings throughout Japan.

## Supplementary Material

CROSS-CULTURAL ADAPTATION AND VALIDATION OF THE JAPANESE TRANSLATION OF THE FUGL-MEYER ASSESSMENT FOR UPPER AND LOWER EXTREMITY SENSORIMOTOR FUNCTION AFTER STROKE
