# Supplementary material for: CROSS-CULTURAL ADAPTATION AND VALIDATION OF THE JAPANESE TRANSLATION OF THE FUGL-MEYER ASSESSMENT FOR UPPER AND LOWER EXTREMITY SENSORIMOTOR FUNCTION AFTER STROKE
Source: J Rehabil Med. 2025 Aug 5;57:43350. doi: 10.2340/jrm.v57.43350 (PMC12340990; doi:10.2340/jrm.v57.43350)
Supplement: CROSS-CULTURAL ADAPTATION AND VALIDATION OF THE JAPANESE TRANSLATION OF THE FUGL-MEYER ASSESSMENT FOR UPPER AND LOWER EXTREMITY SENSORIMOTOR FUNCTION AFTER STROKE [file JRM-57-43350-s1.pdf]

Supplementary material has been published as submitted. It has not been copyedited, or typeset by Journal of Rehabilitation Medicine

Table SI. Percentage agreement (PA) between and within testers for each item of the Fugl-Meyer Assessment of the upper extremity

	Intra-rater agreement		Inter-rater agreement	
	Rater 1	Rater 2	Day 1	Day 2
**A. UPPER EXTREMITY**	Day 1 to 2	Day 1 to 2	Rater 1 to 2	Rater 1 to 2
I. Reflex activity				
Flexors	100	100	100	100
Extensors	100	100	100	100
II. Movement within synergies				
Shoulder retraction	100	100	100	100
Shoulder elevation	100	100	100	100
Shoulder abduction (90°)	100	100	100	100
Shoulder external rotation	100	90	90	100
Elbow flexion	90	90	100	100
Forearm supination	90	100	90	100
Shoulder adduction/internal rotation	90	90	100	100
Elbow extension	90	90	100	100
Forearm pronation	90	90	100	100
III. Movement mixing synergies				
Hand to lumbar spine	100	100	100	100
Shoulder flexion 0°–90°	100	100	100	100
Pronation–supination	90	90	100	100
IV. Movement with little/no synergy				
Shoulder abduction 0°–90°	90	90	100	100
Shoulder flexion 90°–180°	90	100	100	90
Pronation/supination	90	100	100	90
V. Normal reflex activity	80	90	100	90
**B. WRIST**				
Stability at 15° dorsiflexion (90°)	100	100	100	100
Repeated wrist flexion (90°)	90	90	100	100
Stability at 15° dorsiflexion (0°)	100	100	100	100
Repeated wrist flexion (0°)	90	90	100	100
Circumduction	90	100	100	90

**C. HAND**				
Mass flexion	80	80	100	100
Mass extension	100	100	100	100
Grasp				
a. Hook grasp	100	100	100	100
b. Thumb adduction	100	100	100	100
c. Pincer grasp, opposition	100	100	100	100
d. Cylinder grasp	100	100	100	100
e. Spherical grasp	100	100	100	100
**D. COORDINATION/SPEED**				
Tremor	70	80	100	90
Dysmetria	70	80	100	90
Time	100	100	100	100
**H. SENSATION**				
Upper arm, forearm (light touch)	80	80	100	100
Palmar hand (light touch)	100	100	100	100
Shoulder (position)	100	100	100	100
Elbow (position)	100	100	100	100
Wrist (position)	100	100	100	100
Thumb (position)	100	100	100	100
**I. PASSIVE JOINT MOTION**				
Shoulder flexion (0°–180°)	100	100	100	100
Shoulder abduction (0°–90°)	80	80	100	100
Shoulder external rotation	90	80	100	90
Shoulder internal rotation	100	100	100	100
Elbow flexion	90	90	100	100
Elbow extension	100	90	100	90
Forearm pronation	90	80	100	90
Forearm supination	100	100	100	100
Wrist flexion	80	80	100	100
Wrist extension	90	90	100	100
Finger flexion	100	90	100	90
Finger extension	100	100	100	100
**J. JOINT PAIN**				
Shoulder flexion (0°–180°)	80	80	100	100
Shoulder abduction (0°–90°)	80	80	100	100
Shoulder external rotation	100	100	100	100

Shoulder internal rotation	100	100	100	100
Elbow flexion	100	100	100	100
Elbow extension	100	100	100	100
Forearm pronation	100	100	100	100
Forearm supination	100	100	100	100
Wrist flexion	100	100	100	100
Wrist extension	100	100	100	100
Finger flexion	90	90	100	100
Finger extension	100	100	100	100

Table SII. Percentage agreement (PA) between and within raters for each item of the Fugl-Meyer Assessment of the lower extremity

	Intra-rater agreement		Inter-rater agreement	
	Rater 1	Rater 2	Day 1	Day 2
**E. LOWER EXTREMITY**	Day 1 to 2	Day 1 to 2	Rater 1 to 2	Rater 1 to 2
I. Reflex activity				
Flexors	100	100	100	100
Extensors	100	100	100	100
II. Movement within synergies				
Hip flexion	100	100	100	100
Knee flexion	100	100	100	100
Ankle dorsiflexion	100	100	100	100
Hip extension	100	100	100	100
Hip adduction	100	100	100	100
Knee extension	100	100	100	100
Ankle plantar flexion	100	100	100	100
III. Movement mixing synergies				
Knee flexion	90	90	100	100
Ankle dorsiflexion	90	90	100	100
IV. Movement with little/no synergy				
Knee flexion to 90°	80	80	100	100
Ankle dorsiflexion	100	90	90	100
V. Normal reflex activity	100	100	100	100
**F. COORDINATION/SPEED**				
Tremor	80	80	100	100
Dysmetria	80	80	100	100
Time	80	80	100	100
**H. SENSATION**				
Leg (light touch)	**70 (RP)***	**70 (RP)***	100	100
Foot sole (light touch)	**70 (RP)***	**70 (RP)***	100	100
Hip (position)	100	100	100	100
Knee (position)	100	100	100	100
Ankle (position)	100	100	100	100
Great toe (position)	90	90	100	100
**I. PASSIVE JOINT MOTION**				
Hip flexion	80	80	100	100
Hip abduction	90	90	100	100

Hip external rotation	90	90	100	100
Hip internal rotation	100	90	100	90
Knee flexion	100	90	100	90
Knee extension	100	100	100	100
Ankle dorsiflexion	100	100	100	100
Ankle plantar flexion	100	100	100	100
Foot pronation	80	80	100	100
Foot supination	90	90	100	100
**J. JOINT PAIN**				
Hip flexion	100	100	100	100
Hip abduction	100	100	100	100
Hip external rotation	100	100	100	100
Hip internal rotation	100	100	100	100
Knee flexion	100	100	100	100
Knee extension	100	100	100	100
Ankle dorsiflexion	100	100	100	100
Ankle plantar flexion	100	100	100	100
Foot pronation	100	100	100	100
Foot supination	100	100	100	100

*Statistically significant disagreement (absolute value of RP ≥ 0.1 and 95% CI does not include 0) marked in bold

RP: relative position; CI: confidence interval
